# Mid-term follow-up evaluation of a new arthroscopic Broström procedure for chronic lateral ankle instability

**DOI:** 10.1186/s13018-023-03789-3

**Published:** 2023-04-24

**Authors:** Zhongdi Liu, Hao Lu, Yusong Yuan, Zhongguo Fu, Hailin Xu

**Affiliations:** 1 National Center for Trauma Medicine, Ministry of Education Key Laboratory of Trauma and Neural Regeneration, Trauma Medicine Center, Peking University People’s Hospital, Beijing, China; 2 Department of Trauma and Orthopedics, Peking University People’s Hospital, No. 11, South XiZhiMen Street, Beijing, 100044 China

**Keywords:** Chronic ankle instability, Ankle arthroscopy, Ligament repair, Treatment

## Abstract

**Background:**

Chronic lateral ankle instability (CLAI) usually progresses from a previous lateral ankle sprain that was not treated properly. Several procedures have been introduced to address these patients, including open or arthroscopic techniques, the most common of which is the Broström procedure. Here, we describe a new outside-in arthroscopic Broström procedure and its results for treating patients with CLAI.

**Methods:**

Thirty-nine patients (16 male and 23 female; mean age, 35 years [range, 16–60 years]) with CLAI were treated arthroscopically after failing non-operative management. All patients were symptomatic with a combination of recurrent ankle sprains, “giving way,” and avoidance of sports and presented with a positive anterior drawer test upon the physical examination. All patients underwent arthroscopic lateral ligament reconstruction using the new technique. Patient characteristics and pre- and postoperative visual analog scale (VAS), American Orthopedic Foot and Ankle Society Ankle-Hindfoot Scale system (AOFAS), and Karlsson scores were recorded.

**Results:**

The mean AOFAS score increased from 48 (mean 48, range 33–72) preoperatively to 91 (mean 91, range 75–98) at the final follow-up, Karlsson–Peterson and FAAM scores were also significantly improved. Two patients (5.13%) reported superficial peroneal nerve irritation symptoms postoperatively. Three patients (7.69%) complained of mild pain anteroinferior to the lateral ankle.

**Conclusions:**

The arthroscopic outside-in Broström procedure with a single suture anchor was a safe, effective, and reproducible technique for CLAI. Ankle stability resumed with a high clinical success rate. The main complication was injury to the superficial peroneal nerve, which crossed the area of repair.

## Background

Ankle sprain is one of the most common injuries in recreational and professional sports [1]. In addition to athletes, 8% of the general population may also experience persistent symptoms following an initial ankle sprain [2]. Up to 80% of ankle sprain cases are caused by excessive inversion of the ankle, involving the lateral ankle ligament complex [3, 4], especially the anterior talofibular ligament (ATFL), which is the weakest of the lateral ligaments. Although most acute lateral ankle sprains can be successfully treated conservatively, Yeung et al. reported that approximately 20–30% of athletes with previous ankle sprain complain of residual pain and a sense of instability [5].

Chronic lateral ankle instability (CLAI) is a recurrent ankle sprain or a feeling of “giving way” for at least 12 months, resulting from a previous significant ankle injury [6]. “Giving way” is defined as uncontrolled or unpredictable excessive inversion of the ankle during walking or running, which does not lead to acute ankle sprain. CLAI can be further divided into functional ankle instability (FAI) and mechanical ankle instability (MAI). FAI is subjective instability without physical laxity, while MAI is usually caused by lateral ligament injury. Although FAI and MAI may occur independently, reports have indicated that a combination of the two most likely contributes to CLAI [7–9].

If conservative treatments such as physical therapy and fixation with plaster or boots do not work for patients with CLAI, surgical treatment is indicated [10]. A variety of surgical methods have been reported in the literature, including nonanatomic reconstruction, anatomic repair, anatomic reconstruction and arthroscopic surgery [11–13]. Broström proposed an anatomic repair of the ATFL in 1966 [14], which was modified by Gould in 1980 [15] and Karlsson in 1989 [16]. Since then, modified Broström procedures have been most widely used for the treatment of CLAI and shown good clinical results [17]. Arthroscopic repair of the ATFL can be traced back to 1987, which was first reported by Hawkins [18]. Subsequently, a variety of arthroscopic surgical methods for CLAI had been derived, including ATFL ligament reconstruction with and without ATFL remnant repair, all-inside arthroscopic ATFL repair with different suture methods and so on [11–13].

With the development of arthroscopic techniques, there has been increasing popularity in arthroscopic repair of the lateral ligaments and several authors have reported good clinical results with different techniques based on Broström surgery [13, 19–24]. The advantages of arthroscopic repair include less invasiveness and better short-term functional outcome compared with open surgery [25, 26]. In this paper, we describe a novel arthroscopic Broström technique that is simple and easy to handle. We also report a retrospective analysis of the mid-term surgical effect of this technique in the treatment of CLAI.

## Methods

### Patient inclusion criteria and follow-up assessment

All the patients meeting the inclusion criteria were treated with this new arthroscopic Broström procedure between January 2016 and June 2018. The inclusion criteria were as follows: (1) age ≥ 16 years; (2) recurrent inversion ankle sprains or repeated “giving way” during daily or recreational activities secondary to trauma for at least 1 year; (3) positive anterior drawer test or stress radiographs compared with the uninjured side; (4) magnetic resonance imaging (MRI) showing ATFL damage with or without calcaneofibular ligament (CFL) damage; and (5) unresponsive to physical therapy for over 3 months. Patients were excluded if they had (1) neurovascular disorders, (2) ankle deformity, (3) general joint laxity, (4) previous surgery on the affected ankle, and (5) concomitant lesions of the affected ankle, such as osteochondral lesions of the talus, impingement syndrome, os subfibulare, deltoid ligament injury, peroneal tendon tear, syndesmosis injury, and arthritis.

Patient characteristics were collected and summarized. The patients were followed-up regularly after the operation (once a month within 3 months after surgery, after that, follow-up visits were made every 3 months). The visual analog scale (VAS) score, American Orthopedic Foot and Ankle Society Ankle-Hindfoot Scale system (AOFAS) score, Karlsson–Peterson score, and Foot and Ankle Ability Measure (FAAM) were evaluated by an independent observer preoperatively and at final follow-up to assess the ankle function. The anterior drawer test was performed bilaterally to determine the stability of the affected ankle. Postoperative complications were recorded.

This study was approved by the institutional review board of our institution (NCT04736238).

### Surgical technique

**Fig. 1:**
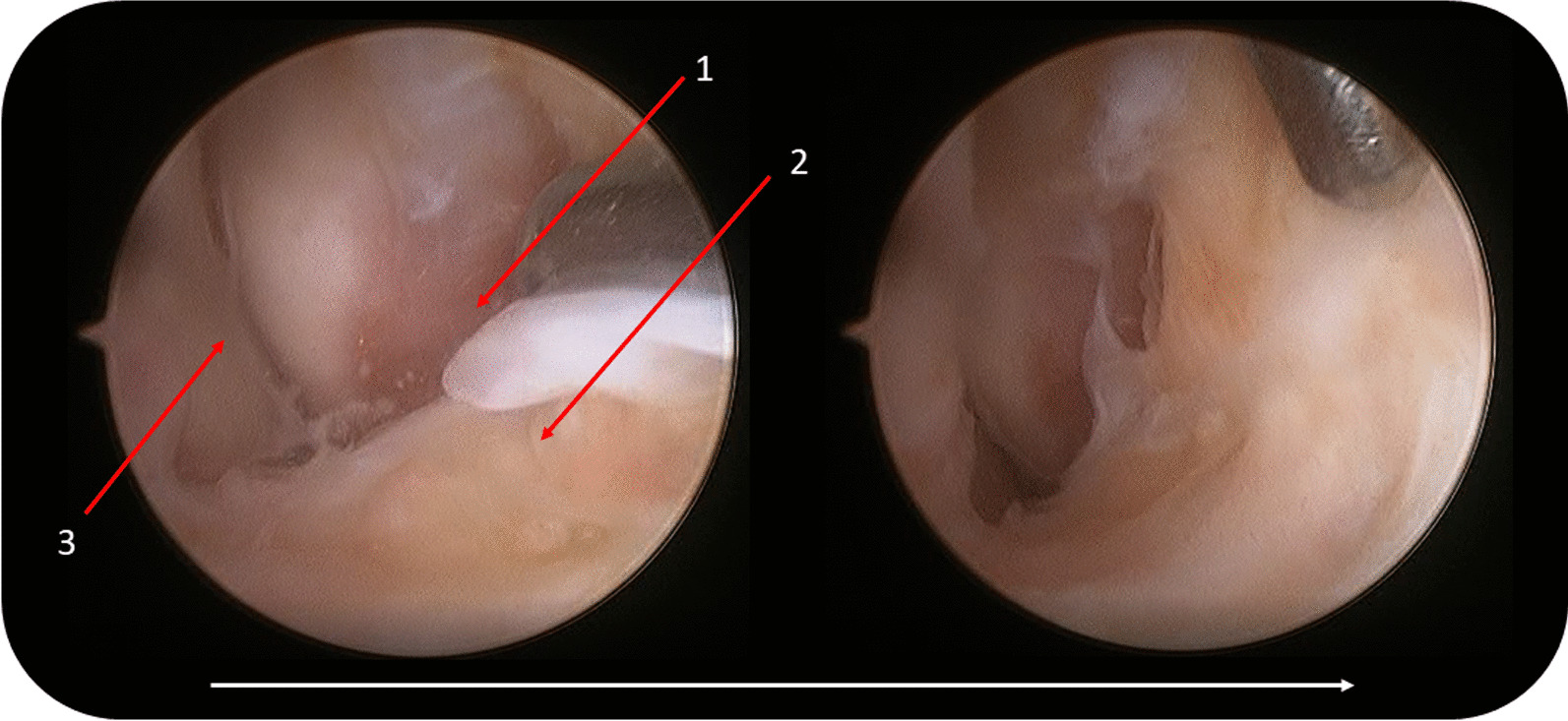
Arthroscopic vision showing the ATFL remnant is separated from the bone with the help of a beaver blade introduced through the anterolateral portal arthroscopic ligament repair (left ankle). (1) Fibula. (2) ATFL remnant. (3) Lateral wall of the talus

Under intrathecal or general anesthesia, the patient was positioned supine with a bump under the ipsilateral hip to internally rotate the ankle to facilitate the procedure. A thigh tourniquet was then applied. A medial midline portal was used for arthroscopic viewing, which was lateral to the tibialis anterior tendon. A 2.7 mm, 30°arthroscope was introduced via this portal. An anterolateral working portal was located anterior to the lateral corner of the talus trochlea. Debridement of lateral gutter was performed with a shaver, and ATFL was evaluated to confirm the presence of remnant fibers of the ATFL (Fig. 1).

**Fig. 2:**
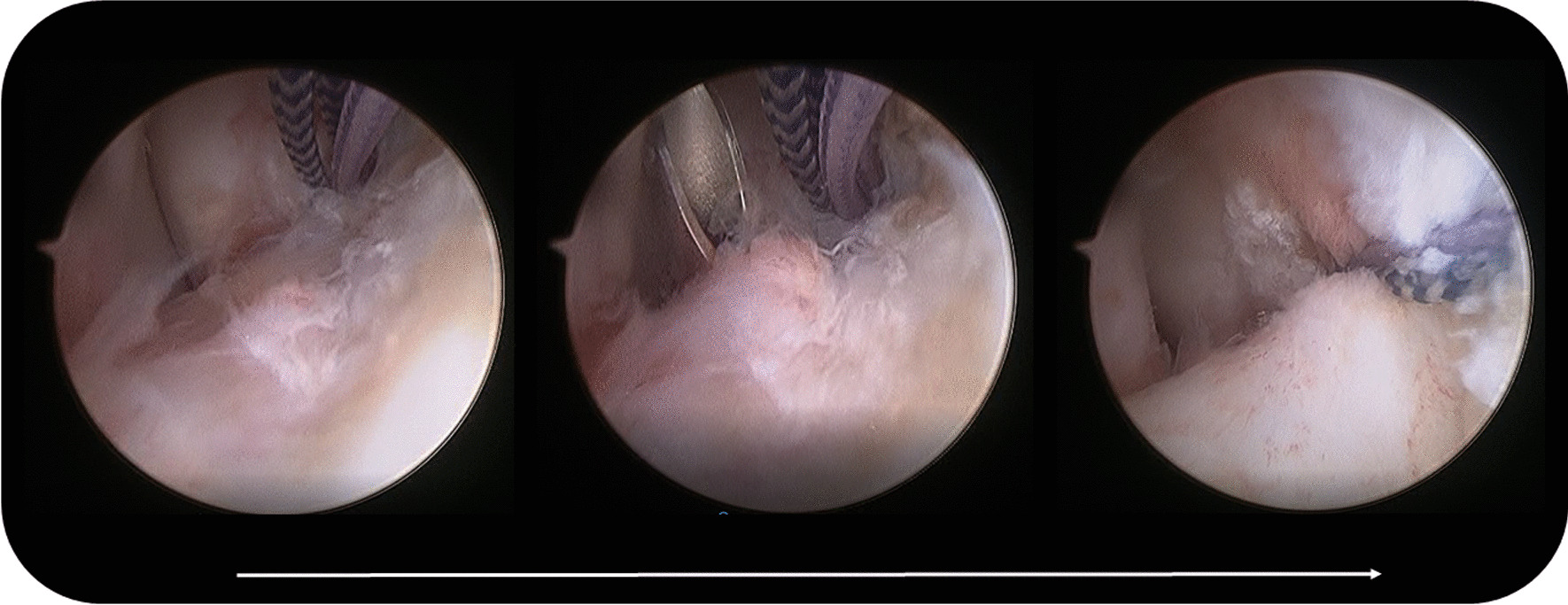
Arthroscopic vision showing anchor introduction, needle used as a suture lasso and reconstructed ligament

**Fig. 3:**
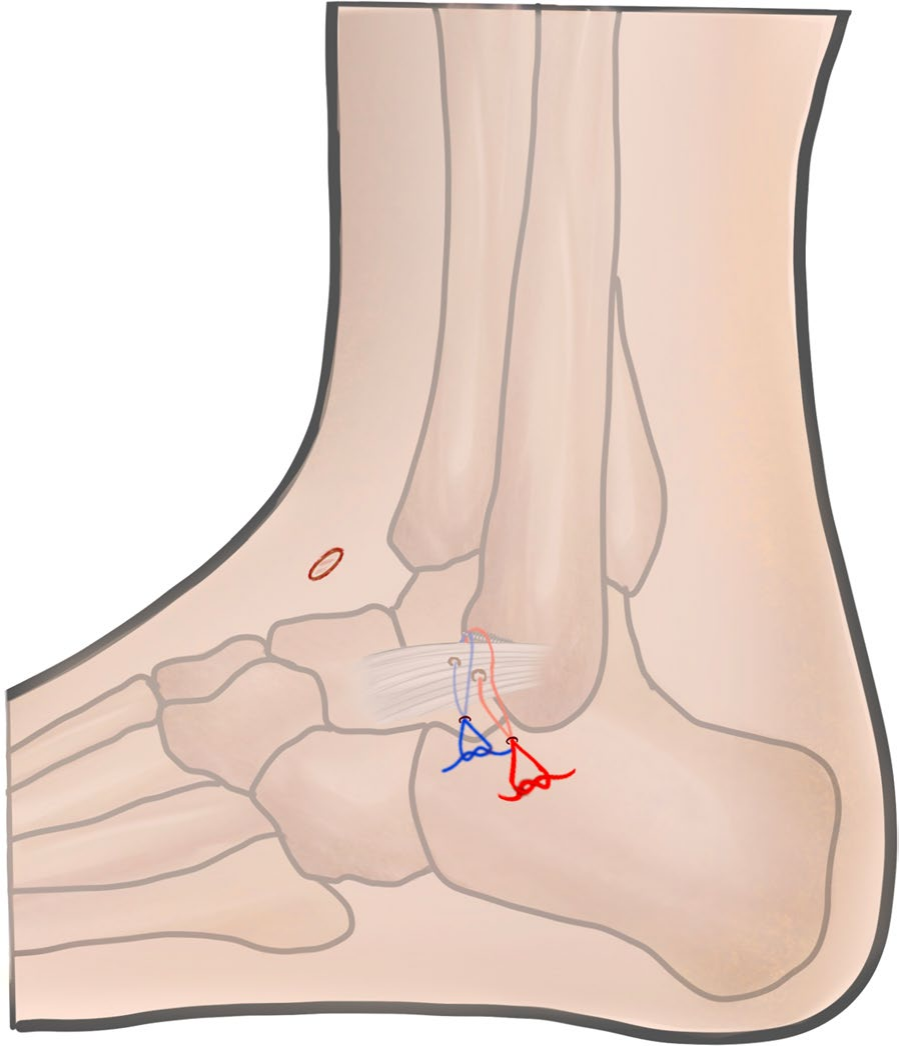
Location of arthroscopic lateral portals and anchor suture for ligament reconstruction

**Fig. 4:**
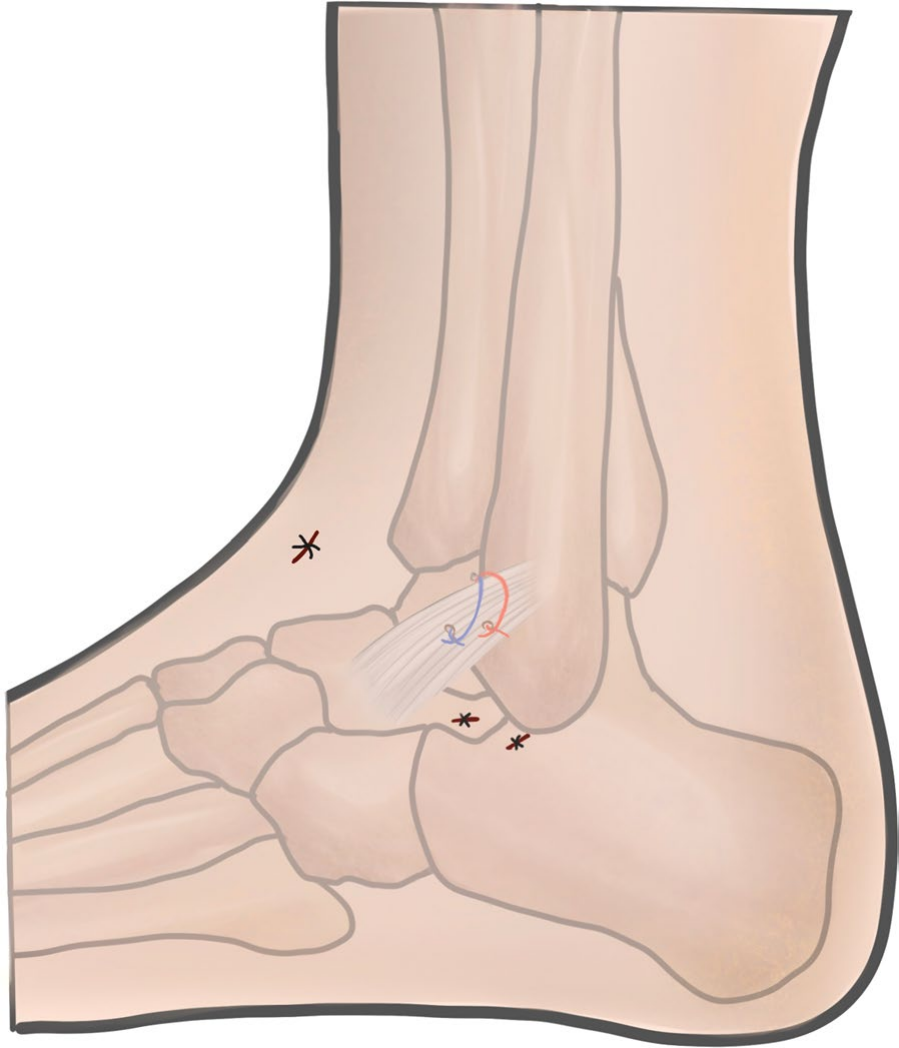
Diagram of the ankle joint after completion of arthroscopic procedure with a single suture anchor

The fibular origin of the ATFL was identified distal to the anterior inferior tibiofibular ligament (AITFL) and proximal to the fibular obscure tubercle. One anchor (2.9 mm Lupine BR, preloaded with double orthocord sutures, DePuy Synthes) was predrilled and inserted at the center of the fibular origin of the ATFL through the anterolateral portal. A 14-gauge cannulated needle, used as a suture lasso, was inserted percutaneously from the anterior aspect of the distal fibula in the “safe zone” into the lateral gutter and penetrated the ATFL (Fig. 2). A PDS II suture was passed through the needle into the lateral gutter and pulled out from the anterolateral portal using a grasper. One limb of the first set of orthocord sutures was tied by PDS II, and the opposite end of PDS II was pulled. Thus, PDS II leads one limb of the orthocord sutures to penetrate the ATFL and exit through the skin at the penetration point of the needle. 2 mm incision was made at the penetration point of the needle. The subcutaneous tissue between the incision and anterolateral portal was bluntly spread to the ankle capsule to decrease the risk of nerve entrapment and skin dimpling. The other limb of the first set of orthocord sutures was grabbed subcutaneously into the incision. The same procedure was repeated with the second set of orthocord sutures, in which the skin penetration point was 5 mm anterosuperior to the first one. There should be a sufficient distance between the two sutures in the ATFL (Fig. 3). Both sets of ultrabraid sutures were tied with the ankle held in a slightly everted and neutral dorsiflexion position. Anterior drawer and inversion stress examinations were performed to confirm adequate stabilization. The incisions were then closed using standard methods (Fig. 4).

After the operation, the ankle was immobilized with a short leg cast to avoid ankle plantarflexion for 2 weeks followed by a lace-up gauntlet ankle brace for another 10 weeks. Progressive weight bearing in a boot-walker was usually tolerated, and ankle dorsiflexion exercise was allowed from the second day after surgery. At 4 weeks, proprioceptive training, ankle plantar flexion exercise, and peroneal muscle strengthening were started. Running and swimming were allowed at 3 months, and high-contact sports were allowed at 6 months postoperatively.

### Statistical analysis

Statistical analysis was performed using SPSS software (version 19.0; SPSS Inc., Chicago, IL, USA). Patient characteristics and outcomes are presented as median, mean, and range. The *p* values were based on the paired *t*-test (normal distribution) and the Wilcoxon signed-rank test (non-normal distribution). Statistical significance was set at *p* < 0.05.

## Results

A total of 39 patients (39 ankles) with CLAI were included, 23 females and 16 males with a mean age of 35 years (range, 16–60 years). No patient loss was observed at the final follow-up. The mean body mass index (BMI) was 22 (range, 16–29) kg/m^2^. The patient characteristics are summarized in Table 1. The arthroscopic Broström procedure was performed by a single surgeon on all patients. During the arthroscopic procedure, all patients were diagnosed with isolated avulsion of the ATFL from the fibula.

**Table 1:** Patient characteristics (*N* = 39)

Patient characteristics	
Age (years)^a^	35 (16–60)
Sex^b^	
Male	16 (41.03%)
Female	23 (58.97%)
BMI (kg/m^2^)^a^	22 (16–29)

^a^Values are given as the mean, with the range in parentheses

^b^Percentage of the total number of patients included

At follow-up, the mean postoperative VAS, AOFAS, Karlsson–Peterson, FAAM Activities of Daily Living (ADL), and FAAM SPORT scores were 1.3 (range, 1–3), 91 (range, 75–98), 88 (range, 77–98), 90 (range, 85–95), and 71 (range, 56–91), respectively. All results showed a significant improvement over the preoperative data. The preoperative and postoperative outcomes and comparisons are presented in Table 2.

**Table 2:** Comparison of VAS, AOFAS, Karlsson–Peterson and FAAM score before and after operation

Items	Preoperation	Postoperation	*p* values
VAS	5.7 (3–8)	1.3 (1–3)	< .001
AOFAS	48 (33–72)	91 (75–98)	< .001
Karlsson–Peterson	52 (33–71)	88 (77–98)	< .001
FAAM ADL	68 (45–82)	90 (84–95)	< .001
FAAM SPORT	44 (28–56)	71 (56–90)	< .001

The mean surgery time was 44 min (range, 35–60 min). No patient had recurrent ankle instability at the final follow-up. Thirty-three patients (84.6%) returned to full activity at the preinjury level, whereas the other 6 patients changed to non-contact sports such as running, cycling, and swimming due to fear of reinjury. The mean follow-up duration was 22 months (mean, 22, range 11–33) months.

Three patients (7.7%) reported complications after surgical procedure. Two (5.13%) patients had superficial peroneal nerve (SPN) irritation symptoms, postoperatively. After treatment with neurotrophic drugs, the symptoms of one patient gradually alleviated, whereas the symptoms of the other patient persisted. Three (7.69%) patients (including patients with SPN irritation symptoms) complained of discomfort anteroinferior to the lateral ankle, which was related to weather change and excessive activity. There were no postoperative complications such as wound infection or venous thrombosis of the lower extremity in any of the 39 patients in this study.

## Discussion

The most important contribution of this study is providing a novel method for the arthroscopic repair of ATFL with a single sutured anchor and a modified Broström procedure. The follow-up results obtained at a mean of 22 months demonstrated excellent outcomes.

The ATFL and CFL are the most frequently involved ligaments in lateral ankle injuries. The ATFL is more prone to injury, which is reported to be 2 to 3.5 times higher than the failure of the CFL [27, 28]. The classic Broström procedure is defined as the repair of the ATFL and CFL [14]. While evolving modified surgical techniques for lateral ankle instability focus on the importance of restoring the ATFL, current practices vary in terms of whether the CFL repair is performed during lateral ligament surgery [29]. Some surgeons suggest that repair of the CFL is necessary based on biomechanical and clinical outcome data, which indicated that the CFL is responsible for resistance to inversion during dorsiflexion in the neutral state, and acts as a stabilizer of the subtalar joint during plantarflexion [30]. Thus, injuries to the CFL contribute to lateral instability of the ankle, leading to significant decreases in rotational stiffness and peak torque, substantial changes in contact mechanics of the ankle, increased varus of the talus and calcaneus, and medial calcaneal displacement [31]. However, some other authors found no significant differences in patients who underwent the repair of ATFL only compared to both ATFL and CFL, arguing that the role of CFL in maintaining ankle stability is limited; therefore, repairing the CFL is not recommended [32].

The inferior extensor retinaculum (IER) has also been previously used to augment lateral ankle ligament reconstruction. IER is an aponeurotic structure, which starts at the lateral surface of the calcaneus, crosses the subtalar joint, and surrounds the tendons of the anterior compartment in continuation with the sural fascia [33]. It could be a Y-shaped or X-shaped structure with strong correlations to the intermediate dorsal cutaneous nerve. The Broström–Gould procedure, involving IER in lateral ankle ligament repair, can be considered as a two-step technique. First, the ATFL is surgically repaired, and in the second step, the IER is attached to the lateral malleolus. With fixation of the IER, the repair of the ATFL is augmented, and it is thought to provide a stronger lateral stabilizer to the ankle [34, 35]. However, some recent anatomical studies confirmed that the IER is a weak band of tissue that will probably not add significant strength to ankle stability. Postoperative follow-up indicated that clinical and radiographic outcomes were not significantly different based on whether or not IER reinforcement was performed. Therefore, isolated ligament reconstruction without IER may be sufficient to restore ankle stability [33].

It has been reported that the clinical outcomes between ATFL remnant repair and non-repair were not relevant when performing an all-inside arthroscopic Brostrom–Gould procedure for CLAI [36], however, in spite of not performing the surgical repair of CFL or IER, the postoperative stability of the ankle joint was effectively restored in this study. Therefore, we believe that the ATFL repair with this outside-in arthroscopic technique could effectively restore the stability of the ankle joint.

However, arthroscopic invention also has some problems, including higher complexity, longer surgical time and more complications. The most common complications were: neuritis of the superficial peroneal nerve, residual instability, persistent lateral pain, prominent suture knots, superficial infections, venous thrombosis and distal fracture of the fibula [25, 26]. In our study, a complication rate of 7.7% was observed, neurological complaints were observed in 5.1% of patients, and only in one case the neurological symptoms persisted until follow-up. The transient neurological symptoms after the operation may be traction injury to nerve during the establishment of the arthroscopic channel, while the persistent neurological symptoms may be nerve compression caused by the knotting of sutures. The occurrence of these complications is usually related to the experience of the operator, differences in arthroscopic techniques, and evaluation criteria. Based on previous clinical experience, we designed this new arthroscopic outside-in technique, which offers patients a minimally invasive reconstruction alternative to the traditional open and other arthroscopic Broström techniques. Additionally, this is an easy to perform procedure, that reduces the interference of the normal structure on the lateral ankle joint, thereby reducing the likelihood of nerve irritation and wound complications.

The limitations of this study include small number of patients recruited for the surgical procedure and a lack of control group of patients that underwent an open ATFL repair. Additionally, the functional outcomes were subjectively reported by the patients at the final postoperative visit for both pre- and postoperative scores, wherein bias may have influenced the outcomes. Therefore, prospective and comparative studies are needed for similar patient populations.

The clinical relevance of this study is the description of an arthroscopic outside-in repair method of the ATFL with a single suture anchor, which offers an effective repair method for chronic lateral ankle instability. Advantages of this technology include the benefit of a minimally invasive approach and the possibility of an arthroscopic ankle joint examination for simultaneously addressing intra-articular pathological entities.

## Conclusions

It is safe and effective to treat chronic lateral ankle instability with this new outside-in Broström procedure under arthroscopy. In the improved Broström operation, we emphasize on the correct selection of indication for the reconstruction of anatomical ligaments. The main complication of this technique is injury to the superficial peroneal nerve, which passes through the operative area. However, the recovery rate of the ankle joint stability was high. Taken together, this technique is a good option for most patients with chronic lateral ankle instability.

## Data Availability

The datasets are available from the corresponding author on reasonable request.

## Abbreviations

ADL: Activities of daily living
AITFL: Anterior inferior tibiofibular ligament
ATFL: Anterior talofibular ligament
AOFAS: American Orthopedic Foot and Ankle Society Ankle-Hindfoot Scale system
BMI: Body mass index
CFL: Calofibular ligament
CLAI: Chronic lateral ankle instability
FAAM: Foot and Ankle Ability Measure
FAI: Functional ankle instability
IER: Inferior extensor retinaculum
MAI: Mechanical ankle instability
MRI: Magnetic resonance imaging
VAS: Visual analog scale
